# Transcriptomics Provides Novel Insights into the Regulatory Mechanism of IncRNA HIF1 A-AS1 on Vascular Smooth Muscle Cells

**DOI:** 10.21470/1678-9741-2022-0260

**Published:** 2023-09-19

**Authors:** Jin Yang, Zhiqiang Gong, Junjie Dong, Haotian Li, Bing Wang, Kaili Du, Chunqiang Zhang, Lingqiang Chen

**Affiliations:** 1 Department of Orthopaedics, The First Affiliated Hospital of Kunming Medical University, Yunnan, People's Republic of China

**Keywords:** Apoptosis, Hypoxia Inducible Factor 1, RNA Sequence Analysis, Thoracic Aortic Aneurysm, Vascular Smooth Muscle Cells

## Abstract

**Introduction:**

Thoracic aortic aneurysm is a potentially fatal disease with a strong genetic contribution. The dysfunction of vascular smooth muscle cells (VSMCs) contributes to the formation of this aneurysm. Although previous studies suggested that long non-coding ribonucleic acid (RNA) hypoxia inducible factor 1 α-antisense RNA 1 (HIF1A-AS1) exerted a vital role in the progression and pathogenesis of thoracic aortic aneurysm, we managed to find a new regulatory mechanism of HIF1A-AS1 in VSMCs via transcriptomics.

**Methods:**

Cell viability was detected by the cell counting kit-8 assay. Cell apoptosis was assessed by Annexin V-fluorescein isothiocyanate/propidium iodide double staining. Transwell migration assay and wound healing assay were performed to check the migration ability of HIF1A-AS1 on VSMCs. The NextSeq XTen system (Illumina) was used to collect RNA sequencing data. Lastly, reverse transcription-quantitative polymerase chain reaction confirmed the veracity and reliability of RNA-sequencing results.

**Results:**

We observed that overexpressing HIF1A-AS1 successfully promoted apoptosis, significantly altered cell cycle distribution, and greatly attenuated migration in VSMCs, further highlighting the robust promoting effects of HIF1A-AS1 to thoracic aortic aneurysm. Moreover, transcriptomics was implemented to uncover its underlying mechanism. A total of 175 differently expressed genes were identified, with some of them enriched in apoptosis, migration, and cell cycle-related pathways. Intriguingly, some differently expressed genes were noted in vascular development or coagulation function pathways.

**Conclusion:**

We suggest that HIF1A-AS1 mediated the progression of thoracic aortic aneurysm by not only regulating the function of VSMCs, but also altering vascular development or coagulation function.

## INTRODUCTION

An aneurysm results from the walls of a blood vessel weaken, causing localized dilatations of the supra-diaphragmatic aorta^[1]^. Aneurysm growth is concentrated with dissection (tearing) or rupture of the aortic wall. Aneurysms may form in any blood vessel of our body, but most commonly in the aorta. The aorta is mainly divided into the thoracic aorta and the abdominal aorta. Thoracic aortic aneurysm (TAA) may involve one or more segments of the thoracic aorta. TAA of the arch or descending thoracic aorta is life-threatening if undiagnosed or neglected as aneurysms expand. Based on the diameter of the aorta and its suspected causes, drug intervention or even ascending aortic replacement may be necessary^[2]^. Nevertheless, patients surviving surgery are more likely to suffer from serious complicating diseases, such as myocardial infarction, renal failure, stroke, neurological deficits, and paraplegia^[3]^. At present, the effective treatment and prevention of TAA are still challenging and limited due to their uncertain pathogenesis. Therefore, revealing the molecular mechanism for the TAA is indispensable for developing effective treatment.

The aortic media mainly consists of vascular smooth muscle cells (VSMCs), which are the main source of extracellular matrix proteins such as collagen and elastin^[4]^.To a great extent, VSMCs determine the biological properties of the aortic wall, where they are of importance in maintaining the normal physiological function of blood vessels. The dysfunction of VSMCs is considered to be an important cause for the formation of TAA^[5]^.

Non-coding ribonucleic acids (RNAs) mainly consist of short/small RNAs, circular RNAs, and those greater than 200 bases, who are called long non-coding RNAs (lncRNAs)^[6,7]^. Although the biological function of IncRNAs have not been thoroughly investigated, numerous IncRNAs have been proved as powerful regulators of diverse human diseases^[8,9]^. Hypoxia inducible factor 1 a(HIF1A) are transcription factors that are activated in response to decreased oxygen availability in the cellular environment. LncRNA hypoxia inducible factor 1 α-antisense RNA 1 (HIF1A-AS1), as one of three kinds of antisense RNA of HIF1A, is located on the antisense strand of HIF1A of human chromosome 14, and the length of mature body is 652 nt^[10]^.

HIF1A-AS1 may exert a vital part in the occurrence and development of some cardiovascular diseases, especially TAA. Our previous study discovered that Clopidogrel inhibited apoptosis and facilitated proliferation in palmitic acid (PA)-treated human vascular endothelial cells by suppressing the mediator HIF1A-AS1^[11]^. Serum exosomes and exosomal HIF1A-AS1 expression level could act as potential biomarkers for atherosclerosis^[12]^. Accumulating evidence provides compelling arguments supporting the involvement of HIF1A-AS1 in the pathogenesis of TAA. It was found that HIF1A-AS1 regulated the proliferation and apoptosis of VSMCs *in vitro* and that the expression of HIF1A-AS1 in serum of TAA patients was upregulated compared with normal control (NC), which might contribute to the pathogenesis of TAA^[13]^. HIF1A-AS1 was found to be upregulated in TAA patient serum via a hierarchical cluster analysis. Silence of HIF1A-AS1 could decrease apoptosis and promoted viabilities of VSMCs induced by PA treatment^[14]^. The abovementioned piece of evidence revealed HIF1A-AS1 is of great importance in the progression of TAA. In addition, the mechanism of HIF1A-AS1 on regulating TAA have been investigated as well. HIF1A-AS1 was reported to be involved in intracranial aneurysms by regulating VSMC proliferation through the upregulation of transforming growth factor beta 1 (TGFβ1)^[15]^. Zhang et al. suggested that HIF1A-AS1 regulated the cell function of VSMCs by regulating let-7g/apoptotic protease activating factor-1 (APAF-1) axis, resulting in development of TAA^[16]^.

Although the role of HIF1A-AS1 in VSMCs have been studied, we managed to find a new regulatory mechanism of HIF1A-AS1 in VSMCs via transcriptomics. In our current study, using a lentivirusbased overexpression system, we found that overexpressing HIF1A-AS1 greatly promoted the apoptosis, observably altered cell cycle distributions, and markedly reduced migration of VSMCs. These data suggested that this IncRNA precisely mediated VSMCs function and owned robust promoting potential to TAA. Additionally, transcriptomics was implemented to explore the underlying mechanism. By setting a strict threshold, we discovered 175 differently expressed genes (DEGs) that may have contributed to this phenomenon. Bioinformatics results revealed most of them enriched in apoptosis, migration, and cell cycle-related pathways. Intriguingly, some DEGs were noted in vascular development or coagulation function pathways. Reverse transcription-quantitative polymerase chain reaction (RT-qPCR) was finally resorted to confirm the veracity and reliability of RNA-sequencing results. In view of this, we suggested that HIF1A-AS1 mediated the progression of TAA by not only regulating the function of VSMCs, but also altering vascular development or coagulation function. Abovementioned findings manifested the crucial role played by HIF1A-AS1 in VSMCs. Our study may provide new clinical ideas for the treatment of TAA.

## METHODS

### Ethics Statement

The approval for experiments was obtained from the Animal Experiment Ethics Committee of Kunming Medical University (approval No. KMMU2021016).

### Cell Line and Treatments

Human aortic VSMCs were purchased from the Shanghai Cell Bank of the Chinese Academy of Sciences (Shanghai, China). The cells were cultured in Dulbecco’s modified Eagle’s medium (Gibco), which contained 10% fetal bovine serum (Gibco) in a 5% CO_2_ incubator at 37°C. When the cell density was > 80%, we washed the cells twice with sterilized phosphate-buffered saline (PBS). Subsequently, 0.25%o trypsin was added to dissociate the cell-cell contacts. After centrifugation (1000 r/min, five minutes), the cells were resuspended in complete medium with 10% fetal bovine serum for further use.

### Plasmid Construction, Lentivirus Package, and Transfection

HIF1A-AS1 cardiovascular diseases was amplified and subcloned into the pLVX-Puro 1.0 empty plasmid using the restriction sites for EcoRI and BamHI (Thermo Scientific). About 2 µg of plasmids were mixed with the lentivirus packaging plasmids pHelper 1.0, pHelper 2.0, and Opti-MEM according to the previous standard protocol^[17]^. Subsequently, VSMCs were infected with 20 multiplicity of infection lentivirus for 24 hours and then incubated in fresh medium. Finally, the cells were washed after 24 hours. The sequences of the cloning primers are listed in Table 1.

**Table 1:** Primer used in reverse transcription-Quantitative polymerase chain reaction.

Gene/shRNA	Forward primer (5'-3')	Reverse primer (5'-3')
IFI27	AATCGCCTCGTCCTCCATAGC	TCGCAATGACAGCCGCAATG
SERPINB2	CCGAGTGAAGCGATGTGGAAC	GGTAGGTAGTGGAGCAGGGATTC
TXNIP	TGCCACCACCGACTTATACTGAG	GCCTGCTGACCACCTCCTAC
S100A7	CTGCTGACGATGATGAAGGAGAAC	GCTTGTGGTAGTCTGTGGCTATG
KRT1	TTCCCTTACTCTACCTTGCTCCTAC	CCACCACCTCCTCCACTGC
FRY	GCTGTTCGTGAGGAGGAGGAC	GCAAGGATGGCTGAGAAGAAGG

FRY=FRY microtubule binding protein; IFI27=interferon alpha inducible protein 27; KRT1=keratin 1; S100A7=S100 calcium binding protein A7; SERPINB2=serpin family B member 2; shRNA=short hairpin ribonucleic acid;TXNIP=thioredoxin interacting protein

### Apoptosis Detection

Apoptosis was assessed by Annexin V-fluorescein isothiocyanate (FITQ/propidium iodide (PI) double staining (KeyGEN BioTECH, Nanjing, China) according to the manufacturer’s protocol, followed by flow cytometry analysis (BD Pharmingen, San Diego, California, United States of America). VSMCs (5.0 × 10^5^/well, 1 ml) were plated in 6-well plates and then washed in PBS to remove the impurities. The cells were resuspended in staining buffer containing 5 µl Annexin V-FITC and 10µl PI for 15 minutes in the dark. Cells were added 300 µl binding buffer and then analyzed by flow cytometry within one hour.

### Cell Cycle Assay

The cell cycle assay was performed as previously described with a slight change^[18]^. In brief, cells were seeded into 96-well plates and then harvested (1 x 10^6^ cells per group) 48 hours later. After centrifugation for three minutes, the cells were collected and fixed with 70% ethanol at 4°C. After 0, 24, 48, and 72 hours, 10 µl cell counting kit-8 solution (Beyotime, Shanghai, China) was added into each well. The cells were cultured for another 0.5 hour before use. The optical density value was captured by microplate reader (Bio-Rad, Hercules, California, United States of America) at 450 nm.

### Transwell Invasion

Transwell invasion assay was performed according to the manufacturer’s instructions. Briefly, chambers were assembled in 24-well plates 8 urn pore transwell inserts (BD Falcon, Franklin Lakes, New Jersey, United States of America), which were coated with 50 µl Matrigel (diluted 1:4 in serum-free media). Treated cells (1×10^5^) were added to the upper chamber medium. Invaded cells on the underside of the inserts were fixed with 4%o paraformaldehyde and stained with 0.1%o crystal violet. Images were captured using a stereo microscope (Leica, Wetzlar, Germany). The cells were counted under the TS100 microscope.

### Wound Healing Assay

VSMCs (5 × 10^5^ cells) were seeded in a 6-well plate for 48 hours and plated on coverslips, and then allowed to reach confluence. Cells were assessed for confluency as a monolayer via light microscopy before the initiation of the wound-healing assay. Scratches were made through a 96-pin tool (Woundmaker), according to the protocol provided by the manufacturer. The cells were washed three times with PBS to remove cell debris and cultured in fresh serum-free media for 12 hours in 37°C, 5% CO_2_ incubator. Images of the wound were taken at 0,24, and 48 hours at x 40 magnification, and the extent of wound size was measured using IMAGEJ software in three wells per group.

### RNA Extraction and Sequencing

Total RNA was extracted from prepared cells using TRIzol reagent (Invitrogen; Thermo Fisher Scientific, Inc.). The RNA was further purified with two phenol-chloroform (1:1) (Beijing Solarbio Science & Technology Co., Ltd., Beijing, China) treatments for 15 minutes at 4°C. Then the cells were treated with RQ1 DNase (Promega, Madison, United States of America) for 30 minutes at 4°C to remove deoxyribonucleic acid. The quality and quantity of the purified RNA were assessed by measuring the absorbance at 260 and 280 nm, and the A260:A280 ratio using a Smartspec Plus Spectrophotometer (Bio-Rad Laboratories, Inc., Hercules, California, United States of America). The integrity of RNA was confirmed by 1.5% agarose gel electrophoresis. 10 µg of total RNAs from each sample were applied to RNA-seq library preparation by a Balancer NGS Library Preparation kit (Gnomegen, San Diego, California, United States of America). Polyadenylated messenger RNAs (mRNAs) were purified and concentrated with oligo (dT)-conjugated magnetic beads (Invitrogen; Thermo Fisher Scientific, Inc.), according to the manufacturer’s protocol. The libraries were prepared using the purified mRNAs through the TruSeq Stranded Total RNA LT Sample Prep Kit (Illumina, Inc., San Diego, California, United States of America). The NextSeq XTen system (Illumina) was used to collect RNA sequencing data.

### Bioinformatic Analysis

The functions of those identified DEGs were annotated by applying gene ontology (GO) annotation software (http://david.abcc.ncifcrf. gov/home.jsp) and Kyoto Encyclopedia of Genes and Genomes (KEGG) pathway database (http://www.genome.jp/kegg).

### Reverse Transcription-Quantitative Polymerase Chain Reaction

To validate the mRNA-seq data, real-time quantitative PCR (qPCR) was applied for detecting some mRNAs randomly selected from those DEG. The primers used are listed in Table 1. The qPCR conditions were as follows: pre-denaturation at 95°C for one minute, 40 cycles of denaturing at 95°C for 15 seconds, annealing at 60°C for 30 seconds, and elongation at 72°C for 40 seconds. The results were quantified by the 2 ^−△△CT^ method^[19]^.

### Statistical Analysis

The results are presented as the means ± standard error of mean or standard deviation for the indicated number of experiments. Three independent experiments were performed. Quantitative data were compared using the x^2^ test. Statistical significance was evaluated by Student’s *t*-test and analysis of variance assay. *P*-values ≤ 0.05 were considered to be statistically significant.

###  Availability of Data and Materials

The data sets analyzed during this study are available from the NCBI public repository (accession number: GSE202078).

## RESULTS

### Establishment of a Lentivirus-Based Overexpression System for HIF1A-AS1 in VSCMs

**Fig. 1:**
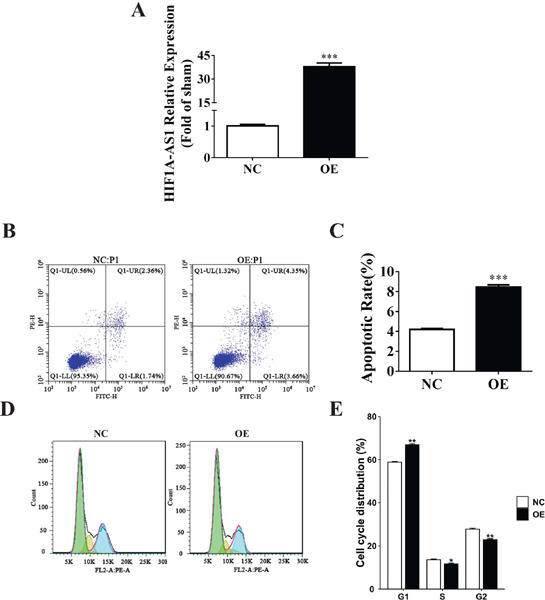
Hypoxia inducible factor 7 α-antisense RNA 1 (HIF1A-AS1) facilitated apoptosis and disrupted cell cycle. (A)Reverse transcription-quantitative polymerase chain reaction verified the successful construction of the vector (B) Two groups of cells were treated for assessment of apoptosis using Annexin V-fluorescein isothiocyanate staining coupled with flow cytometry Each group had three parallel controls. The upper left, lower left, upper right, and lower right quadrants represent necrotic, normal, late apoptotic, and early apoptotic events, respectively. (C) Total percentage of apoptotic dendritic cell in each treatment group were quantified with data presented as the mean ± standard deviation of three independent experiments. (D) Cell cycle distribution was strikingly altered in the HIF1A-AS1 overexpressing cells. Representative images from three independent experiments were shown. (E) Quantification of cell cycle distribution in the HIF1A-AS1 overexpressing vascular smooth muscle cells and its control cells from three independent experiments. *P<0.05; **P<0.005; ***P<0.001. FITC-H=fluorescein isothiocyanate-height; FL2-A:PE-A=ratio of the area under the curve of the fluorescence channel 2 and the phycoerythrin channel; NC=normal control; OE=overexpressed; PE-H=phycoerythrinheight; Q1-LL=lower left quadrant; Q1 -LR=lower right quadrant; Q1 -UL=upper left quadrant; Q1-UR=upper right quadrant.

VSCMs have been widely used in the studies on TAAs progression and cancer-related signaling pathways. We overexpressed (OE) the full length of HIF1A-AS1 gene stably in VSCMs via a lentivirusbased expression system (or ptt5-HIF1 A-AS1). Correspondingly, empty plasmid was transfected to VSMCs as control under the same condition. In order to assess the transfection efficiency of VSCMs specific to this IncRNA, we measured the expression level of HIF1A-AS1 in those two groups of cells. The efficacy of HIF1A-AS1 overexpression, as assessed by RT-qPCR, was shown by about 38-fold increase at transcriptional level (Figure 1A). It manifested a successful establishment of the lentivirus-based HIF1A-AS1 overexpression system.

### HIF1A-AS1 Facilitated Apoptosis and Disrupted Cell Cycle

We wondered whether HIF1A-AS1 overexpression caused programmed cell death, as previously reported^[3,13]^. So, we counted the number of apoptotic cells in both HIF1A-AS1 OE and NC groups. By staining prepared cultures with Annexin-V conjugated and FITC, a notably 1-fold increase was observed in VSMCs apoptosis in OE groups, suggesting the elevated level of apoptosis induced by this IncRNA. Particularly, early and late apoptotic cells increased about 55% and 46%, respectively (Figures 1B and 1C). These results suggested that HIF1A-AS1 greatly promoted VSMCs apoptosis.

In addition, we resorted to flow cytometry to uncover the distribution of specific phases of cell cycle when HIF1A-AS1 stably OE. In the OE group was observed an appreciably increasing proportion (66.95%o) of G1 phase compared with the NC group (58.91%)). The cell proportion in S phase decreased about 2.01% in the OE group. Subsequently, it was found that the OE group contained a greatly decreasing proportion of G2 phase cells (22.89%) compared with the NC group (27.69%) (Figures 1D and 1E). These findings indicated that HIF1A-AS1 disrupted cell cycle of VSMCs.

### HIF1A-AS1 Attenuated Migration Ability of VSMCs

**Fig. 2:**
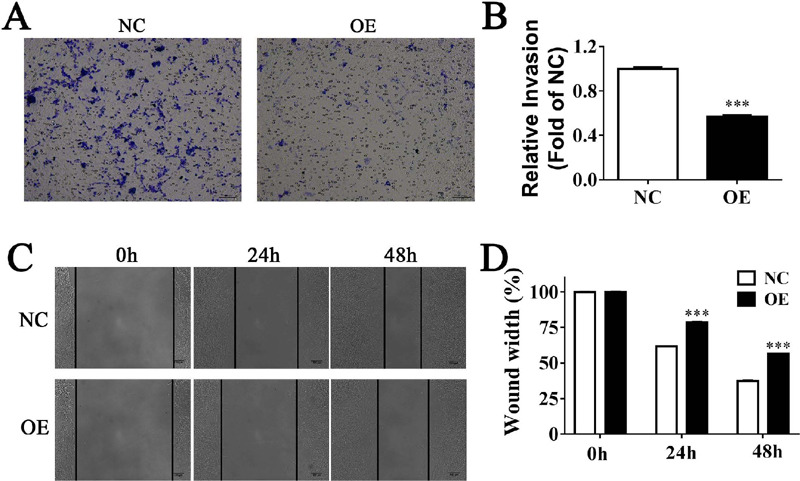
Hypoxia inducible factor 1α-antisense RNA 7 (HIF1A-AS1) attenuated migration ability of vascular smooth muscle cells (VSMCs). (A & B) Relative cell migrations were examined in six groups of cells by transwell assay Quantitative analysis of wound healing was from 3 fields. (C&D) Wound healing assays showed that HIF1A-AS1 obviously decreased migration ability of VSMCs. Values shown are mean ± standard deviation from three independent experiments. ***P<0.001. NC=normal control; OE=overexpressed.

Transwell migration assay and wound healing assay were performed to check the migration ability of HIF1A-AS1 on VSMCs. The result of transwell migration assay showed that cell invasion ability in OE groups declined about 43.3%o compared with NC groups, which indicated the observably weaken migratory ability of PA-treated VSMCs (Figures 2A and B). In addition, the negative effect of HIF1A-AS1 on VSMCs’ migration was further confirmed by scratch wound healing assay. As vividly revealed in Figures 2C and 2D, VSMCs migrated for a shorter distance in the pore plate extracted from HIF1A-AS1 OE cells than in that from NC groups. These data demonstrated that HIF1A-AS1 overexpression attenuated the migration ability of VSMCs.

### RNA-seq Summary

**Fig. 3:**
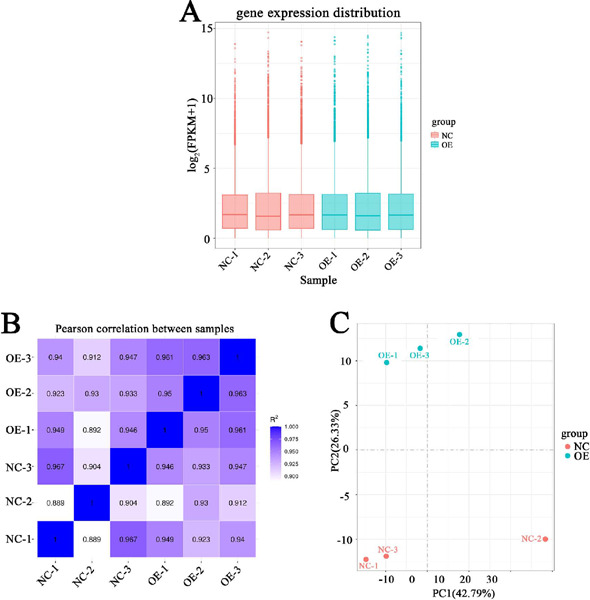
Summory of ribonucleic acid-sequencing results. (A) Boxplot showed the fragments per kilobase per million mopped fragments (FPKM) distribution of genes from the 6 samples. (B) The Pearson correlation among 6 samples was displayed. (C) The principal component (PC) analysis among those 6 samples was displayed. NC=normal control; OE=overexpressed.

Transcriptomics was applied to comprehensively investigate HIF1A-AS1 -mediated transcriptional regulation. Firstly, six complementary deoxyribonucleic acid (cDNA) libraries, named NC-1, NC-2, NC-3, OE-1, OE-2, and OE-3, were prepared from NC and OE cells, with two biological replicates. The six cDNA libraries were then sequenced using the NextSeq X Ten system. After removing adaptor sequences and low-quality sequencing reads, we obtained a total of about 0.266 billion high-quality reads (clean reads), corresponding to about 44.3 million sequence reads per sample. The error rates in the process of sequencing were about 0.03%, and the proportion of Q20 (the Phred value > 20) was more than 96% in those six samples (Table 2). In addition, > 86%o of clean reads were successfully mapped against the current human reference genome. The detail results for each sample were presented in Table 3. Then, we calculated the expression values of all genes in each sample, and no significant difference could be found among those groups (Figure 3A).The results of the Pearson correlation data were presented in Figure 3B. The principal component analysis results (Figure 3C) indicated that it was rather different between the NC and OE groups. Abovementioned pieces of evidence confirmed the stability and reliability of RNA-sequencing data.

**Table 2:** Small RNA-sequencing results.

Sample	Raw reads	Clean reads	Error rate (%)	Q20
NC-1	41412020	40155610	0.03	97.08
NC-2	51109242	49902022	0.03	96.94
NC-3	43489732	41773152	0.03	97.03
OE-1	54070380	52628356	0.03	96.9
OE-2	43074282	42466858	0.03	97.08
OE-3	39767632	39073052	0.03	96.71

NC=normal control; OE=overexpressed; RNA=ribonucleic acid

**Table 3:** Clean reads mapped to the current human reference genome.

Sample	Total reads	Total map	Unique map	Multi map
NC-1	40155610	37779605 (94.08%)	35446470 (88.27%)	2333135(5.81%)
NC-2	49902022	46629452 (93.44%)	43399073 (86.97%)	3230379 (6.47%)
NC-3	41773152	39058343 (93.5%)	36598491 (87.61%)	2459852 (5.89%)
OE-1	52628356	49332472 (93.74%)	46289831 (87.96%)	3042641 (5.78%)
OE-2	42466858	39983009 (94.15%)	37433129 (88.15%)	2549880 (6.0%)
OE-3	39073052	36497946 (93.41%)	34285629 (87.75%)	2212317 (5.66%)

NC=normal control; OE=overexpressed

### Identification of Differently Expressed Genes

**Fig. 4:**
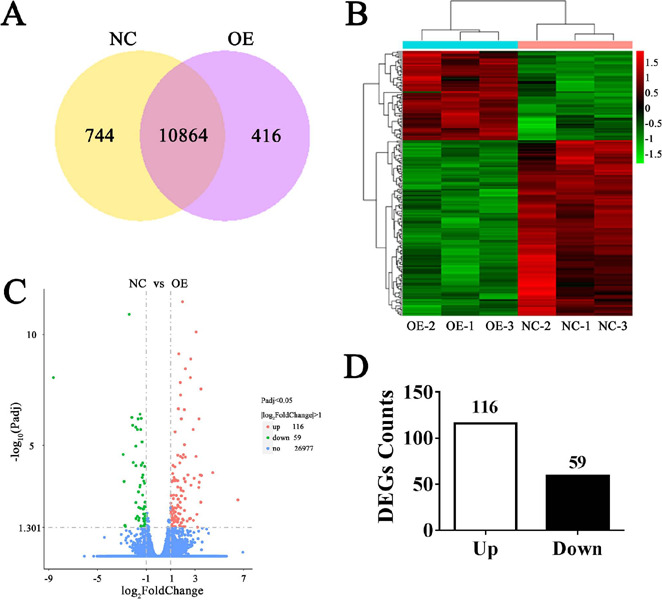
identification of differently expressed genes (DEGs). (A) Venn diagrams of all identified genes in those two comparisons. Heat map (B) and volcano plot (G) of DEGs expression profiles between OE and sham-OE. (D) The number of upregulated and downregulated DEGs between OE and NG. NG=normal control; OE=overexpressed.

The co-expression Venn diagram showed the number of genes that were uniquely or collectively expressed in each group. This suggested that the OE group identified 11,280 genes, of which 416 were uniquely expressed. And the NC group identified 11,608 genes, and 744 of them were uniquely expressed. There were 10,864 genes co-expressed in both two groups (Figure 4A). The identified genes in those six samples were collected to generate heat map plots of the total gene intensities, which helped us to vividly appreciate the detailed alterations. The results showed that HIF1A-AS1 overexpression globally mediated genes expression in VSMCs (Figure 4B). DEGs were determined using the following stringent criteria: Padj < 0.05 and |log_2_ FC (fold change) > 1 .Volcano plots were generated to intuitively reflect the significant up or downregulated genes (Figure 4C). It revealed a total of 175 DEGs identified in NC *vs.* OE group, of which 116 were upregulated and 59 were downregulated (Figure 4D).

### Functional Analysis of DEGs

**Fig. 5:**
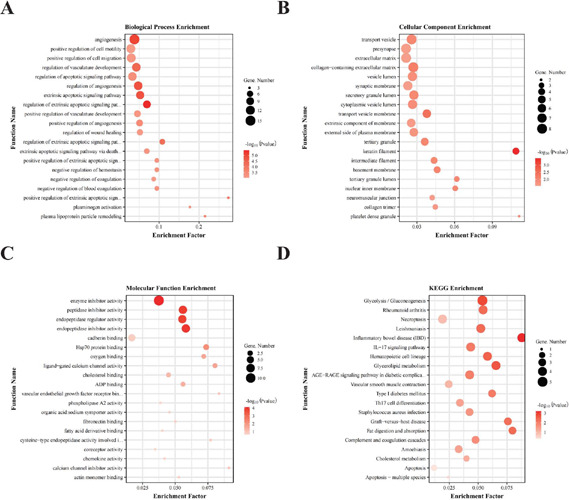
Functional analysis of differently expressed genes (DEGs). The biological process (A), cellular component (B), and molecular function (C) in gene ontology pathway enrichment analysis. (D) Kyoto Encyclopedia of Genes and Genomes (KEGG) pathway enrichment analysis of DEGs from overexpressed vs. normal control groups. ADP=adenosine diphosphate; AGE-RAGE=advanced glycation end products-receptor for advanced glycation end products; IL- 17=interleukin 17; Th 17=T helper cell 17.

It was obvious that OE of HIF1A-AS1 could induce large-scale changes in VSMCs mRNA composition, which has translated into its pivotal roles in TAAs. Consequently, bioinformatics analysis was performed to identify the vital functions in which all DEGs may involve. GO analysis results showed that in terms of biological process (Figure 5A), some DEGs were enriched in some apoptosis or migration-related pathways, such as "regulation of extrinsic apoptotic signaling pathway (GO:2001236)", "extrinsic apoptotic signaling pathway (GO:0097191)", "positive regulation of cell migration (GO:0030335)", "positive regulation of cell motility (GO:2000147)", and so on. It indicated that the DEGs induced by HIF1A-AS1 might serve a regulatory role in the apoptosis and migration of VSMC cells. Some DEGs were annotated into "pyridine nucleotide biosynthetic process (GO:0019363)", "nicotinamide nucleotide biosynthetic process (GO:0019359)", and "positive regulation of cell division (GO:0051781)" (data not shown), implying its role in regulatory cell cycle. Intriguingly, we found that some DEGs were enriched in vasculature development or coagulationrelated pathways, such as "angiogenesis (GO:000152)", "regulation of angiogenesis (GO:0045765)", "negative regulation of blood coagulation (GO:0030195)", "negative regulation of hemostasis (GO: 1900047)", and so on. This implied the potential role HIF1A-AS1 in regulating TAA. Cellular component analysis of GO enrichment (Figure 5B) revealed that those DEGs were mainly located in membrane, vesicle, and extracellular matrix parts. Molecular function (Figure 5C) analysis of GO enrichment showed that some DEGs were enriched in calcium-related pathways, such as "calcium-release channel activity (GO:0015278)", "ligand-gated calcium channel activity (GO:0099604)", "calcium channel inhibitor activity (GO:0019855)", and "Cadherin binding (GO:0045296)", and others were enriched in "cholesterol binding (GO:0015485)", "vascular endothelial growth factor receptor binding (GO:0005172)", "actin monomer binding (GO:0003785)", and "fibronectin binding (GO:0001968)" pathways et aI.

In addition, KEGG enrichment analysis was implemented to further uncover the underlying mechanism. Some DEGs were significantly enriched in those signal pathways that regulated vessel function, such as "vascular smooth muscle contraction (hsa04270)", "cholesterol metabolism (hsa04979)", and "steroid biosynthesis (hsa00100)", while others were associated with"apoptosis-multiple species (hsa04215)","apoptosis (hsa04210)"et al. (Figure 5D).

### HIF1A-AS1 Regulates the Expression of those DEGs at the Transcriptional Level

**Fig. 6:**
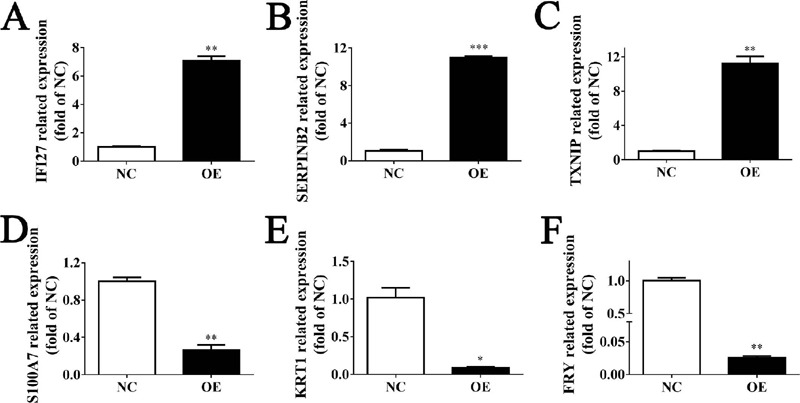
Reverse transcription-quantitative polymerase chain reaction (RT-qPCR) verified the veracity and reliability of ribonucleic acid (RNA)-sequencing data. (A-C) RT-qPCR validated the messenger RNA (mRNA) expression level of three upregulated genes, named IFl27 (A), SERPINB2 (B), and TXNIP (C). (D-F) RT-qPCR validated the mRNA expression level of three down regulated genes, named S100A7 (D), KRT1 (E), and FRY (F). *P<0.05; **P<0.005; ***P<0.001. FRY=FRY microtubule binding protein; IFI27=interferon alpha inducible protein 27; KRT1=keratin 1; NC=normal control; OE=overexpressed; S100A7=S100 calcium binding protein A7; SERPINB2=serpin family B member 2; TXNIP=thioredoxin interacting protein.

To confirm the veracity and reliability of RNA-sequencing results, some genes were randomly selected from those 175 DEGs to investigate the mRNA levels. The expression levels of six genes, including three upregulated proteins and three downregulated proteins, were measured by RT-qPCR. The six proteins were named interferon alpha inducible protein 27 (IFI27), serpin family B member 2 (SERPINB2), thioredoxin interacting protein (TXNIP), S100 calcium binding protein A7 (or S100A7), keratin 1 (or KRT1), and FRY microtubule binding protein (or FRY). Upon qPCR-based quantification, we observed that mRNA expression level of IFI27, SERPINB2, and TXNIP were significantly upregulated (Figures 6A,6B,6C), while the other three genes were downregulated (Figures 6D,6E, 6F). These data were in accordance with RNA-sequencing results and highlighted the reliability and robustness of our detection method. The observation also implies that HIF1A-AS1 globally altered the mRNA level of VSMCs. However, further investigations were still required to explore other mechanisms that might be involved in the HIF1A-AS1 -mediated regulation of mRNA level.

## DISCUSSION

TAA is regarded as a life-threatening vascular disease to human health. The dysfunction of VSMCs contributes to the formation of TAA^[20]^. Previous studies suggested HIF1A-AS1 exerted a vital role in VSMCs and might be involved in the pathogenesis of TAAs. It was found that brahma-related gene 1 (BRG1) expression was significantly higher in TAAs, and its depletion may downregulate the expression of HIF1A-AS1. They further found that suppression of HIF1A-AS1 inhibited apoptosis and promoted proliferation in VSMCs. Based on these findings, they suggested that the interaction between BRG1 and HIF1-AS1 markedly contributed to cell function of VSMCs *in vitro*^[3]^. HIF1A-AS1 was significantly upregulated in the serum of TAA patients. Silence of this IncRNA protected PA-induced cell apoptosis in VSMCs^[13,14]^. It was observed that HIF1A-AS1 expression levels in blood were increased in patients with intracranial aneurysms when compared with healthy people. HIF1A-AS1 overexpression also promoted TGFβ1 expression and inhibited VSMC proliferation. It was finally concluded that HIF1A-AS1 might participate in intracranial aneurysms by regulating VSMC proliferation through upregulating TGFβ1^[15]^. HIF1A-AS1, which was upregulated in TAA tissues, suppressed proliferation, induced apoptosis, and reduced the expression of extracellular matrix proteins in VSMCs through let-7g/APAF-1 axis^[16]^. Abovementioned studies indicated that HIF1A-AS1 induced apoptosis and suppressed proliferation. These pieces of evidence, together, establish that HIF1A-AS1 has a robust pro-apoptotic function and can promote the progression of TAA. Although previous studies identified that HIF1A-AS1 affected the cell function of VSMCs, we managed to uncover its underlying mechanism in a whole new perspective. Considering the multifaceted nature of its functions, we opine that HIF1A-AS1 overexpression might have induced large-scale changes in the cellular transcriptome. In the present study, we investigated the role of HIF1A-AS1 in VSMCs and its underlying mechanism. It was found that HIF1A-AS1 significantly facilitated apoptosis, disrupted cell cycle, and attenuated migration ability of VSMCs, implying its potential role in the pathogenesis and progression of TAA. Furthermore, transcriptomics revealed that HIF1A-AS1 globally mediated the mRNA level of VSMCs and those 175 DEGs identified by RNA-sequencing might contribute to this phenomenon. Interestingly, TGFβ1 and APAF-1, which were reported to be upregulated by HIF1A-AS1 overexpression^[15,16]^, were not listed in those DEGs. Individual differences may be responsible for this unusual phenomenon.

Bioinformatics analysis revealed that, in terms biological process, some DEGs were enriched in apoptosis or migration-related pathways. It meant HIF1A-AS1 changed the expression level of some apoptosis and migration-related genes, which was the reason why HIF1A-AS1 successfully regulated VSMC apoptosis and migration. Some DEGs also associated with the metabolic process of nucleic acid, which explained the regulatory role of HIF1A-AS1 on cell cycle of VSMCs. Intriguingly, we observed a global change in the mRNA levels related to vasculature development or coagulation function. TAA is characterized as the weakened walls of a blood vessel. Relieving chronic disseminated intravascular coagulation might be beneficial for the treatment of TAA^[21^,^22]^.These evidences indicated that HIF1A-AS1 mediated the progression of TAA by not only regulating the function of VSMC cells, but also influencing vascular development or coagulation function by affecting the expression of some related genes.

Also, those identified DEGs were mainly located in membrane, vesicle, and extracellular matrix parts. This isn’t surprising considering the eclectic HIF1A-AS 1 functions and significant cellular changes it can introduce. Molecular function analysis showed some DEGs were enriched in calcium-related pathways. Calcium ions can bind with many coagulation proteins, and these ion-protein interactions played a vital role in the function of coagulation cascade^[23,24]^. Some DEGs were enriched in "cholesterol binding (GO:0015485)", "vascular endothelial growth factor receptor binding (GO:0005172)" "actin monomer binding (GO:0003785)", and "fibronectin binding (GO:0001968)" pathways. Non-high-density-lipoprotein cholesterol is thought to be useful for predicting arteriosclerosis^[25]^. Vascular endothelial growth factor is known for its role in promoting angiogenesis^[26,27,28]^. Fibronectins are essential for organ and blood vessel morphogenesis^[29]^. These evidences further suggested that these DEGs possessed some binding capacity related to vascular development and coagulation function. Our study, therefore, supports the previously established tumor-promoting effects of HIF1A-AS1 overexpression in VSMCs.

## CONCLUSION

In conclusion, we demonstrated that HIF1A-AS1 overexpression facilitated apoptosis, altered cell cycle distribution, and suppressed migration in VSMCs through large-scale alterations of mRNA levels. Besides, some DEGs were enriched in vascular development or coagulation function pathways. We suggested that HIF1A-AS1 mediated the progression of TAA by not only regulating the function of VSMC cells, but also altering vascular development or coagulation function by affecting the expression of some related genes. Our study uncovered the underlying regulatory mechanism of HIF1A-AS1 on VSMCs from a new perspective, and this finding may provide new clinical ideas for the treatment of TAA.

## Abbreviations, Acronyms & Symbols

ADP: Adenosine diphosphate
AGE-RAGE: Advanced glycation end products-receptor for advanced glycation end products
APAF-1: Apoptotic protease activating factor-1
BRG1: Brahma-related gene 1
cDNA: Complementary deoxyribonucleic acid
DEGs: Differently expressed genes
FITC: Fluorescein isothiocyanate
FITC-H: Fluorescein isothiocyanate-height
FL2-A:PE-A: Ratio of the area under the curve of the fluorescence channel 2 and the phycoerythrin channel
FPKM: Fragments per kilobase per million mapped fragments
OE: Overexpressed
PA: Palmitic acid
PBS: Phosphate-buffered saline
PC: Principal component
PE-H: Phycoerythrin-height
PI: Propidium iodide
Q1 -LL: Lower left quadrant
Q1-LR: Lower right quadrant
Q1 -UL: Upper left quadrant
Q1-UR: Upper right quadrant
qPCR: Quantitative polymerase chain reaction
RNA: Ribonucleic acid
FRY: FRY microtubule binding protein
GO: Gene ontology
HIF1A: Hypoxia inducible factor 1 α
HIF1A-AS1: Hypoxia inducible factor 1 α-antisense RNA 1
IFI27: Interferon alpha inducible protein 27
IL-17: lnterleukin 17
KEGG: Kyoto Encyclopedia of Genes and Genomes
KRT1: Keratin 1
IncRNAs: Long non-coding RNAs
mRNA: Messenger RNAs
NC: Normal control
RT-qPCR: Reverse transcription-quantitative polymerase chain reaction
S100A7: S100 calcium binding protein A7
SERPINB2: Serpin family B member 2
shRNA: Short hairpin ribonucleic acid
TAA: Thoracic aortic aneurysm
TGFβ1: Transforming growth factor beta 1
Th17: T helper cell 17
TXNIP: Thioredoxin interacting protein
VSMCs: Vascular smooth muscle cells

## References

[1] Nouri A, Autrusseau F, Bourcier R, Gaignard A, L'allinec V, Menguy C (2020). Characterization of 3D bifurcations in micro-scan and MRA-TOF
images of cerebral vasculature for prediction of intracranial
aneurysms. Comput Med Imaging Graph.

[2] Salameh MJ, Black JH 3rd, Ratchford EV (2018). Thoracic aortic aneurysm. Vasc Med.

[3] Wang S, Zhang X, Yuan Y, Tan M, Zhang L, Xue X (2015). BRG1 expression is increased in thoracic aortic aneurysms and
regulates proliferation and apoptosis of vascular smooth muscle cells
through the long non-coding RNA HIF1A-AS1 in vitro. Eur J Cardiothorac Surg.

[4] Mozafari H, Zhou C, Gu L (2019). Mechanical contribution of vascular smooth muscle cells in the
tunica media of artery. Nanotechnology Reviews.

[5] Durdu S, Deniz GC, Balci D, Zaim C, Dogan A, Can A (2012). Apoptotic vascular smooth muscle cell depletion via BCL2 family
of proteins in human ascending aortic aneurysm and
dissection. Cardiovasc Ther.

[6] Hulshoff MS, Del Monte-Nieto G, Kovacic J, Krenning G (2019). Non-coding RNA in endothelial-to-mesenchymal
transition. Cardiovasc Res.

[7] Yue Y, Lin X, Qiu X, Yang L, Wang R (2021). The molecular roles and clinical implications of non-coding RNAs
in gastric cancer. Front Cell Dev Biol.

[8] Liu X, Zhao S, Sui H, Liu H, Yao M, Su Y (2022). MicroRNAs/LncRNAs modulate MDSCs in tumor
microenvironment. Front Oncol.

[9] Yang M, Lu H, Liu J, Wu S, Kim P, Zhou X (2022). IncRNAfunc: a knowledgebase of IncRNA function in human
cancer. Nucleic Acids Res.

[10] Wang YK, Liu CM, Lin T, Fang CY, Yu CC, Yu CH (2020). Inhibition of HIF1A-AS1 impedes the arecoline-induced migration
activity of human oral mucosal fibroblasts. J Formos Med Assoc.

[11] Wang J, Chen L, Li H, Yang J, Gong Z, Wang B (2015). Clopidogrel reduces apoptosis and promotes proliferation of human
vascular endothelial cells induced by palmitic acid via suppression of the
long non-coding RNA HIF1A-AS1 in vitro. Mol Cell Biochem.

[12] Wang Y, Liang J, Xu J, Wang X, Zhang X, Wang W, Chen L (2017). Circulating exosomes and exosomal IncRNA HIF1A-AS1 in
atherosclerosis. Int J Clin Exp Pathol.

[13] Zhao Y, Feng G, Wang Y, Yue Y, Zhao W (2014). Regulation of apoptosis by long non-coding RNA HIF1A-AS1 in
VSMCs: implications for TAA pathogenesis. Int J Clin Exp Pathol.

[14] He Q, Tan J, Yu B, Shi W, Liang K (2015). Long noncoding RNA HIF1A-AS1A reduces apoptosis of vascular
smooth muscle cells: implications for the pathogenesis of thoracoabdominal
aorta aneurysm. Pharmazie.

[15] Xu J, Zhang Y, Chu L, Chen W, Du Y, Gu J (2019). Long non-coding RNA HIF1A-AS1 is upregulated in intracranial
aneurysms and participates in the regulation of proliferation of vascular
smooth muscle cells by upregulating TGF-β1. Exp Ther Med.

[16] Zhang X, Li H, Guo X, Hu J, Li B (2020). Long noncoding RNA hypoxiainducible factor-1 alpha-antisense RNA
1 regulates vascular smooth muscle cells to promote the development of
thoracic aortic aneurysm by modulating apoptotic protease-activating factor
1 and targeting let-7g. J Surg Res.

[17] Cribbs AP, Kennedy A, Gregory B, Brennan FM (2013). Simplified production and concentration of lentiviral vectors to
achieve high transduction in primary human T cells. BMC Biotechnol.

[18] An HJ, Lee CJ, Lee GE, Choi Y, Jeung D, Chen W (2022). FBXW7-mediated ERK3 degradation regulates the proliferation of
lung cancer cells. Exp Mol Med.

[19] Livak KJ, Schmittgen TD (2001). Analysis of relative gene expression data using real-time
quantitative PCR and the 2(-Delta Delta C(T)) method. Methods.

[20] Cesarini V, Pisano C, Rossi G, Balistreri CR, Botti F, Antonelli G (2019). Regulation of PDE5 expression in human aorta and thoracic aortic
aneurysms. Sei Rep.

[21] Cameron SJ, Russell HM, Owens AP 3rd (2018). Antithrombotic therapy in abdominal aortic aneurysm: beneficial
or detrimental?. Blood.

[22] Fukuda N, Shimohakamada Y, Nakamori Y, Tominaga T, Shinohara K, Takahashi T (2002). [Thoracic aortic aneurysm with chronic disseminated intravascular
coagulation treated successfully with orally administered camostat mesilate,
warfarin and aspirin]. Rinsho Ketsueki.

[23] Furie B, Furie BC (1988). The molecular basis of blood coagulation. Cell.

[24] Cheng J, Wang Y, Pan Y, Li X, Hu J, Lü J (2019). Single-molecule nanomechanical spectroscopy shows calcium ions
contribute to chain association and structural flexibility of blood clotting
factor VIII. Biochem Biophys Res Commun.

[25] Ouchi G, Komiya I, Taira S, Wakugami T, Ohya Y (2022). Triglyceride/low-density-lipoprotein cholesterol ratio is the
most valuable predictor for increased small, dense LDL in type 2 diabetes
patients. Lipids Health Dis.

[26] Chiodelli P, Rezzola S, Urbinati C, Federici Signori F, Monti E, Ronca R (2017). Contribution of vascular endothelial growth factor receptor-2
sialylation to the process of angiogenesis. Oncogene.

[27] Laakkonen JP, Lähteenvuo J, Jauhiainen S, Heikura T, Ylä-Herttuala S (2019). Beyond endothelial cells: vascular endothelial growth factors in
heart, vascular anomalies and placenta. Vascul Pharmacol.

[28] Miller B, Sewell-Loftin MK (2022). Mechanoregulation of vascular endothelial growth factor receptor
2 in angiogenesis. Front Cardiovasc Med.

[29] George EL, Baldwin HS, Hynes RO (1997). Fibronectins are essential for heart and blood vessel
morphogenesis but are dispensable for initial specification of precursor
cells. Blood.

